# DNA methylation mechanisms in the maturing and ageing oocyte

**DOI:** 10.1186/s13072-025-00600-x

**Published:** 2025-06-11

**Authors:** Carla Caniçais, Sara Vasconcelos, Fátima Santos, Sofia Dória, C. Joana Marques

**Affiliations:** 1 Genetics Unit, Department of Pathology, Faculty of Medicine University of Porto (FMUP), Porto, 4200-319 Portugal; 2 ICBAS-School of Medicine and Biomedical Sciences, University of Porto, Porto, 4050-313 Portugal; 3 RISE-Health, Faculty of Medicine University of Porto (FMUP), Porto, 4200-319 Portugal; 4 Epigenetics Programme, The Babraham Institute, Cambridge, CB22 3AT UK; 5 Present Address: Altos Labs Cambridge Institute of Science, Cambridge, CB21 6GP UK

**Keywords:** Epigenetics, Oocyte, DNA methylation, Ageing, Genomic imprinting

## Abstract

Oocyte maturation involves both nuclear and cytoplasmic processes that are critical for the acquisition of oocyte competence. Granulosa cells, surrounding the oocyte, play a pivotal role in the maturation process, with mechanisms such as cAMP signaling significantly influencing oocyte development. Epigenetic mechanisms – including DNA methylation and its oxidative derivatives, histone post-translational modifications and chromatin remodeling – interfere with the accessibility of transcription factors to regulatory regions of the genome, such as promoter regions of genes, hence generally regulating gene expression profiles; however, in oocytes, transcription is largely independent of DNA methylation patterns. Here we highlight epigenetic reprogramming events occurring during oocyte development and ageing, focusing on the establishment of gamete-specific epigenetic marks, including DNA modifications at imprinted regions, and age-related epigenetic changes. We focus on the mechanisms of DNA methylation and demethylation during mouse and human oocyte maturation, alongside an exploration of how ageing impacts the oocyte epigenome and its implications for reproductive success. By providing a comprehensive analysis of the role of epigenetics in oocyte development and maturation, this review addresses the importance of comprehending these processes to enhance in vitro fertilization treatments and improve reproductive outcomes.

## Background

Oocyte development and maturation are complex processes culminating in the release of a mature oocyte capable of fertilization. The competence of the oocyte relies on the precise regulation of epigenetic and transcriptional activities during oocyte maturation, which are critical for the successful development of the future embryo. Although current understanding of these molecular mechanisms is still rather limited, particularly in humans, it is well established that epigenetic modifications play a significant role in oocyte competence – a factor that progressively declines with reproductive aging.

This review provides an overview of studies on DNA methylation and its derivative DNA hydroxymethylation, throughout oocyte maturation, in both human and mouse models. It emphasizes the potential impact that these epigenetic modifications have on the biological processes underlying oocyte maturation. Furthermore, we explore the effects of ageing on the oocyte epigenome and how it compromises the competency for fertilization and subsequent embryonic development. By addressing these topics, this review aims to advance the understanding of the intricate interplay between epigenetic modifications, oocyte maturation, ageing and reproductive outcomes.

## Oocyte development and maturation

The oocyte is the largest cell in the human body and possesses unique structural and functional characteristics. Its development begins during fetal life, with ~ 26 000 oogonia present by the 6th week embryogenesis, increasing to ~ 250 000 by the 9th week, at which point they enter meiosis [1]. Oocytes become arrested at the diplotene stage of prophase I, also known as the germinal vesicle (GV) stage - a prolonged arrest termed the dictyotene state. Meiotic resumption occurs at puberty, marked by germinal vesicle breakdown (GVBD), spindle formation orchestrated by activated mitogen-activated protein kinase (MAPK), and progression through metaphase I (MI), culminating in first polar body extrusion. A second arrest occurs at metaphase II (MII), maintained by cytostatic factor (CSF)-mediated stabilization of maturation promoting factor (MPF), and persists until fertilization [2].

From birth to puberty, human oocytes are halted in meiosis I, after which a limited number are cyclically recruited for growth and maturation, with one or two typically reaching the MII stage for ovulation. Oocyte maturation involves both nuclear and cytoplasmatic events essential for acquiring development competence. While nuclear maturation drives meiotic progression, cytoplasmic maturation is equally critical for fertilization and embryogenesis. Remarkably, only a small number of oocytes complete maturation and only one undergoes ovulation, highlighting the complexity of this tightly regulated process.

**Nuclear maturation** encompasses the processes that allow immature oocytes to progress to maturity through modifications of nuclear components. During embryonic development, female germ cells enter meiosis and arrest at the diplotene stage of prophase I, forming GV oocytes. These oocytes contain a large nucleus and display a decondensed chromatin configuration known as the Non-Surrounded Nucleolus (NSN). As the oocyte growths, chromatin condenses around the nucleolus, transitioning to the Surrounded Nucleolus (SN) configuration [3]. This meiotic arrest is maintained by high intra-oocyte levels of molecules such as cyclic adenosine monophosphate (cAMP) and the activity of the natriuretic peptide precursor type C (NPPC)/natriuretic peptide receptor 2 (NPR2) signalling pathway in surrounding granulosa cells (GC) [4]. Upon the luteinizing hormone (LH) surge, the NPPC/NPR2 system is downregulated, leading to the closure of gap junctions between the oocyte and cumulus cells (CC), a reduction in intra-oocyte cAMP levels, and initiation of GV breakdown (GVBD) [5]. Following GVBD, meiotic spindle assembly and chromosomes alignment at MI occur. Homologs chromosomes segregate during anaphase I, and the first polar body is extruded. The oocyte then enters meiosis II, arresting at MII until fertilization.

**Cytoplasmic maturation** involves extensive molecular and structural changes essential for the acquisition of oocyte development competence. These include the activation and degradation of maternal mRNAs and the reorganization of organelles. Because maturing oocytes are transcriptionally silent, they rely on previously stored, translationally repressed mRNAs, which become activated upon meiosis resumption. These transcripts encode proteins involved in spindle formation, maintenance of MII arrest, and mRNA degradation [6]. The activation of maternal mRNAs is regulated by cytoplasmic polyadenylation, involving the extension of the poly(A) tail in the 3´-untranslated region (3´-UTR), mediated by cytoplasmic polyadenylation element (CPE) and CPE-binding proteins [7]. Conversely, translational repression is achieved through deadenylation and mRNA looping [8]. This temporal regulation of maternal transcripts is critical for successful oocyte maturation and early embryonic development.

**Organelle dynamics** also play a vital role. Mitochondria, essential for ATP production, undergo redistribution, changes in morphology and mitochondrial DNA (mtDNA) copy number to meet the energy demands of maturation [9]. Disrupted mitochondrial function and low ATP levels are associated with oocyte quality [10]. The endoplasmic reticulum (ER) is another key organelle, involved in Ca^2+^ storage and release via Ca^2+^-ATPases, storage proteins, and channels [11]. These calcium oscillations post-fertilization are crucial for MII completion, maternal mRNA recruitment, and the block to polyspermy [12, 13]. The Golgi apparatus (GA) is dispersed in GV oocytes but fragments following GVBD, contributing to the formation of cortical granules [14]. These granules migrate to the oocyte cortex and, upon fertilization, release their contents to modify the zona pellucida (ZP), preventing polyspermy and ensuring monospermic fertilization [15].

Granulosa Cells (GCs) play a critical role in supporting oocyte maturation by regulating the exchange of signalling molecules such as cAMP [16, 17]. Two subtypes of GC exist in pre-antral follicles: mural GCs, which line the follicular wall and are steroidogenically active, and Cumulus Cells (CCs), which directly surround the oocyte and form the cumulus-oocyte complex (COC) [18]. Transzonal projections from CCs penetrate the ZP, enabling the supply of factors and regulatory molecules to oocytes. Initially, meiotic arrest is sustained by elevated cAMP and cGMP (cyclic guanosine 3’,5’-monophosphate) levels within GCs and the oocyte. cGMP inhibits phosphodiesterase 3 A (PDE3A), preventing cAMP hydrolysis and maintaining high intra-oocyte cAMP concentrations. Evidence suggests that the oocyte may also produce cAMP independently [2].

Luteinizing hormone (LH) and follicle stimulating hormone (FSH) mediate an increase in cAMP production by activation of mitogen-activated protein kinases (MAPKs), which act on the GCs and promote and increase in the levels of cAMP within the granulosa cell compartment. This leads to reduced intra-oocyte cGMP and cAMP levels and ultimately triggers meiotic resumption [18].

## Epigenetics: a general overview

The process by which a fertilized zygote develops into a mature, complex organism involves epigenetic regulation, which plays a crucial role in cell differentiation, tissue growth and organismal development. Indeed, the term “Epigenetics” has been historically linked to the process of differentiation, from the initial studies by Conrad Waddington on the canalization of development to the work of Robin Holliday on the effects of DNA modifications on gene expression and cellular differentiation [19, 20]. It was only later that the term “Epigenetics” started to include mechanistic information, such as heritability and reversal of epigenetics marks, and the biochemical nature of these marks, pointing to the “nuclear inheritance not based on differences in DNA sequences” [21]. Epigenetic marks comprise chemical modifications that are mitotically and/or meiotically heritable and that do not entail a change in DNA sequence. Instead, they consist of chemical modifications observed in the DNA, such as 5-methylcytosine (5mC) and its oxidative derivatives – 5-hydroxymethylcytosine (5hmC), 5-formylcytosine (5fC) and 5-carboxylcytosine (5caC), and post-translational modifications on the histone tails. These modifications can enhance or repress transcription by modulating chromatin structure and, consequently, accessibility for transcription factor binding [22]. 

Epigenetic regulation is orchestrated by enzymes that function as writers, readers and erasers of these chemical modifications [22]. DNA methylation is catalysed by the DNA methyltransferases (DNMTs), which add a methyl group (-CH_3_) to the fifth carbon of cytosine pyrimidine bases, predominantly within CpG dinucleotides, yielding a 5-methylcytosine (5mC) [23]. These CpG dinucleotides are enriched at promoter regions, where methylation typically leads to transcriptional repression. The DNMTs family comprises enzymes with distinct functions with DNMT1, DNMT3A and DNMT3B exhibiting DNA methyltransferase activity. DNMT1 preferentially methylates hemi-methylated DNA and is primarily responsible for maintaining methylation patterns during semi-conservative DNA replication. DNMT1 interacts with the multidomain protein UHRF1 (ubiquitin-like with PHD and ring finger domains 1), which contains an SRA domain that binds to hemimethylated CpG dinucleotides and is required for maintenance of methylation in vivo [24]. The DNMT3 family of enzymes are responsible for *de novo* methylation of unmethylated DNA, with DNMT3A and DNMT3B being the major enzymes to establish DNA methylation during embryonic development. In contrast, DNMT3L acts mainly in the germ lines, being catalytically inactive due to a deletion of the conserved C-terminal methyltransferase domains; nevertheless, DNMT3L serves as a cofactor of DNMT3A/3B to enhance their methyltransferase activity [25, 26]. Additionally, a newly identified DNA methyltransferase – DNMT3C - has been proposed to act in the male germ line of rodents, methylating repetitive elements and protecting male germ cells from their deleterious activity [27]. 

**Fig. 1:**
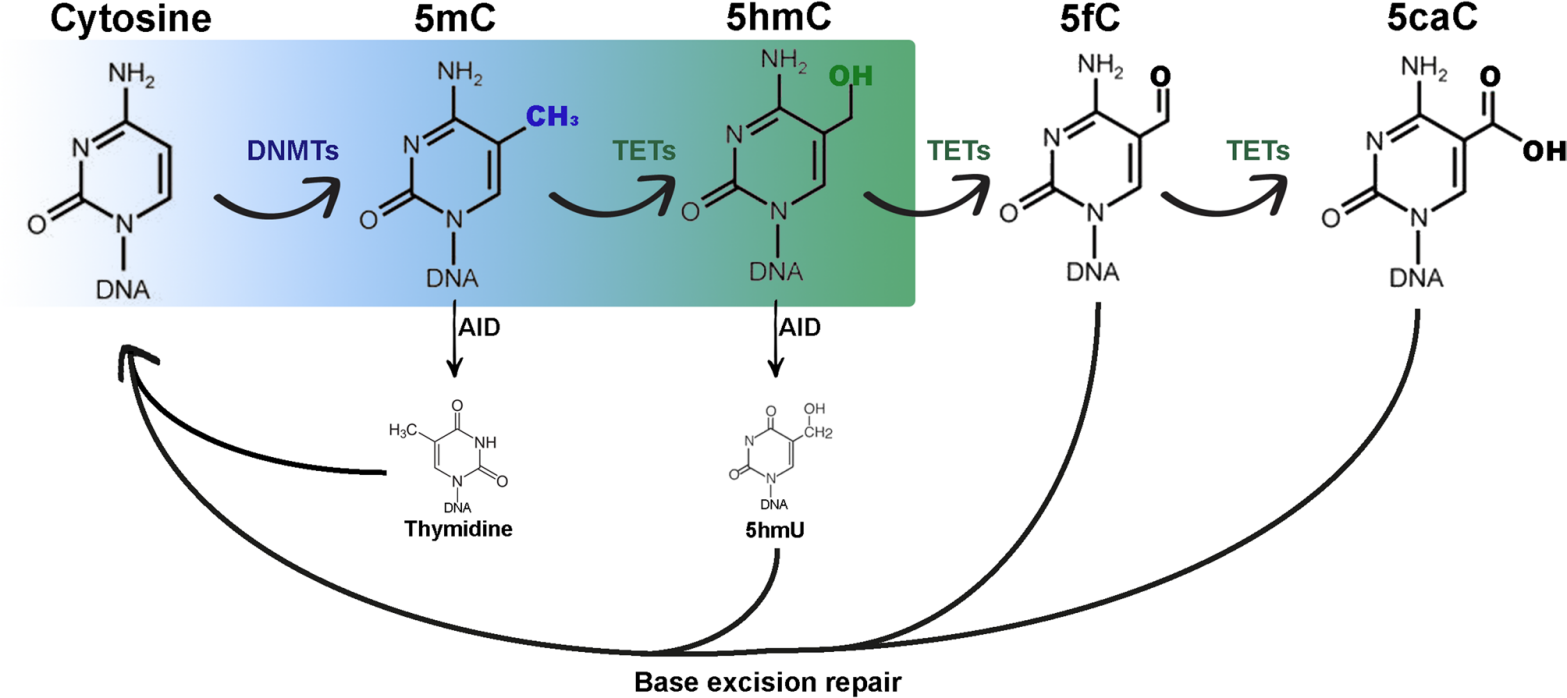
Possible pathways involved in the DNA demethylation process, with representation of the epigenetic regulators and modified bases that are intermediaries in this mechanism. 5mC – 5-methylcytosine; 5hmC – 5-hydroxymethylcytosine; 5fC – 5-formylcytosine; 5caC – 5-carboxylcytosine; 5hmU – 5-hydroxymethyluracil; DNMTs – DNA methyltransferases; TETs – Ten-eleven translocation enzymes; AID – Activation-induced cytidine deaminase

While 5-methylcytosine (5mC) is a major player in epigenetic regulation, another DNA modification, 5-hydroxymethylcytosine (5hmC) was later identified. This modification results from the oxidation of 5mC by the ten-eleven translocation (TET) protein family, specifically, TET1, TET2, and TET3. TET enzymes are Fe(II)/α-ketoglutarate(α-KG)-dependent oxygenases that use molecular oxygen as a substrate to catalyse oxidative decarboxylation of α-KG, generating a reactive high-valent enzyme-bound Fe(IV)-oxo intermediate that converts 5mC to 5hmC [28]. Iterative oxidation of 5hmC by TET enzymes generates 5-formylcytosine (5fC) and 5-carboxylcytocine (5caC), with both modifications being detected in embryonic stem cells [29, 30]. These oxidative derivatives are considered intermediates in the active DNA demethylation pathway, which is thought to involve the thymine-DNA glycosylase (TDG) activity, followed by the base excision repair (BER) [31] (Fig. 1). 

DNA methylation plays an important role in the regulation of oogenesis and in the establishment of maternal imprinting marks. Genomic imprinting is a mammalian mechanism of gene regulation that results in the expression of only one of the parental alleles, leading to parental-of-origin-specific monoallelic expression of imprinted genes [32]. Primordial germ cells (PGCs), the precursors of gametes, undergo erasure of pre-existing somatic imprinting marks to establish gamete-specific imprints. *De novo* methylation at imprinted regions is progressively re-established in female germ cells after birth, whereas in male germ cells it occurs mainly before birth [33]. Germline primary imprints are established during gametogenesis whereas somatic secondary imprints occur during post-implantation development or postnatally, to ensure monoallelic tissue-specific expression. Interestingly, the majority of germline primary imprints are maternally methylated DMRs [34].

**Fig. 2:**
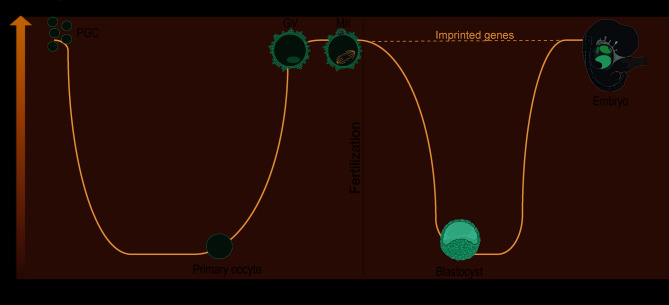
The two major epigenetic reprogramming events that take place during embryogenesis and shape the epigenomic profile of oogenesis and embryogenesis. Global loss of DNA methylation is observed in primordial germ cells (PGCs), followed by gamete-specific acquisition of methylation patterns, including parental imprints and a second-wave of DNA demethylation takes place just hours after fertilization, coinciding with the protamine-to-histone replacement in the paternal genome and the activation of the embryonic genome. This second-wave of demethylation does not perturb previously established parental germline imprinting marks. GV – Germinal vesicle oocyte; MII – Metaphase II oocyte

It is well established that the mammalian epigenome undergoes two major reprogramming events (Fig. 2). The first event occurs in PGCs, where global DNA demethylation takes place as these cells migrate into the genital ridge, resulting in the erasure of maternal and paternal imprinting marks [35]. This is followed by *de novo* methylation, which establishes sex-specific methylation patterns during gametogenesis. The second event occurs after fertilization, characterized by global loss of DNA methylation, although imprinting marks remain unaffected. The paternal pronucleus undergoes active DNA demethylation by the action of TET3 enzyme, a process completed before the first round of DNA replication [36–38]. In contrast, maternal methylation is passively erased during subsequent cleavage divisions, as the oocyte-specific isoform of DNMT1 remains mostly excluded from the nucleus up until the eight-cell stage [39]. DNA methylation of the embryonic genome is then established, beginning before the blastocyst stage and being distinguishable between the inner cell mass (ICM) and the trophectoderm (TE) layer of the blastocyst, with the former showing hypermethylated DNA comparing to the latter [40].

In the following chapters of this review, we will focus on epigenetic events occurring during oocyte maturation and ageing.

## Epigenetic modifications during oocyte maturation

### DNA methylation during oocyte maturation – mouse studies

As mentioned above, PGCs, the precursors of germ cells, undergo genome-wide epigenetic reprogramming by embryonic day 13.5, upon their entry into the gonads, with nearly complete erasure of methylation (less than 10% remaining) [35]. In mouse oogonia, methylation levels remain low until birth, when the oocyte-growing stage begins [41].

Initial studies on mouse oocyte methylomes revealed that fully grown GV oocytes exhibit an average CG methylation level of about 40%, which is less than half of what is observed in the male gamete (~ 90%) [42]. On the other hand, non-growing (NG) oocytes from newborn mice display CG methylation levels as low as 2.3%, suggesting that DNA methylation accumulates during oocyte growth [43]. Notably, a strong bimodal distribution of methylated CpGs was reported in the mouse oocyte, where methylated and unmethylated CpGs were not randomly distributed throughout the genome but rather comprising hypermethylated and hypomethylated domains [44]. The majority of CpG sites were found within these domains, predominantly in hypomethylated regions. Only a minority of CpG sites were located within CpG islands (CGI), which were mostly hypomethylated, with methylated CGIs primarily found within transcription units. Similar findings were reported in another study, which identified a higher percentage of methylated CpG sites in regions with low CpG density (~ 50%) [42]. Furthermore, several studies revealed a correlation between transcription and DNA methylation, with most of the *de novo* methylation being attributable to transcriptional events [42, 44, 45].

**Fig. 3:**
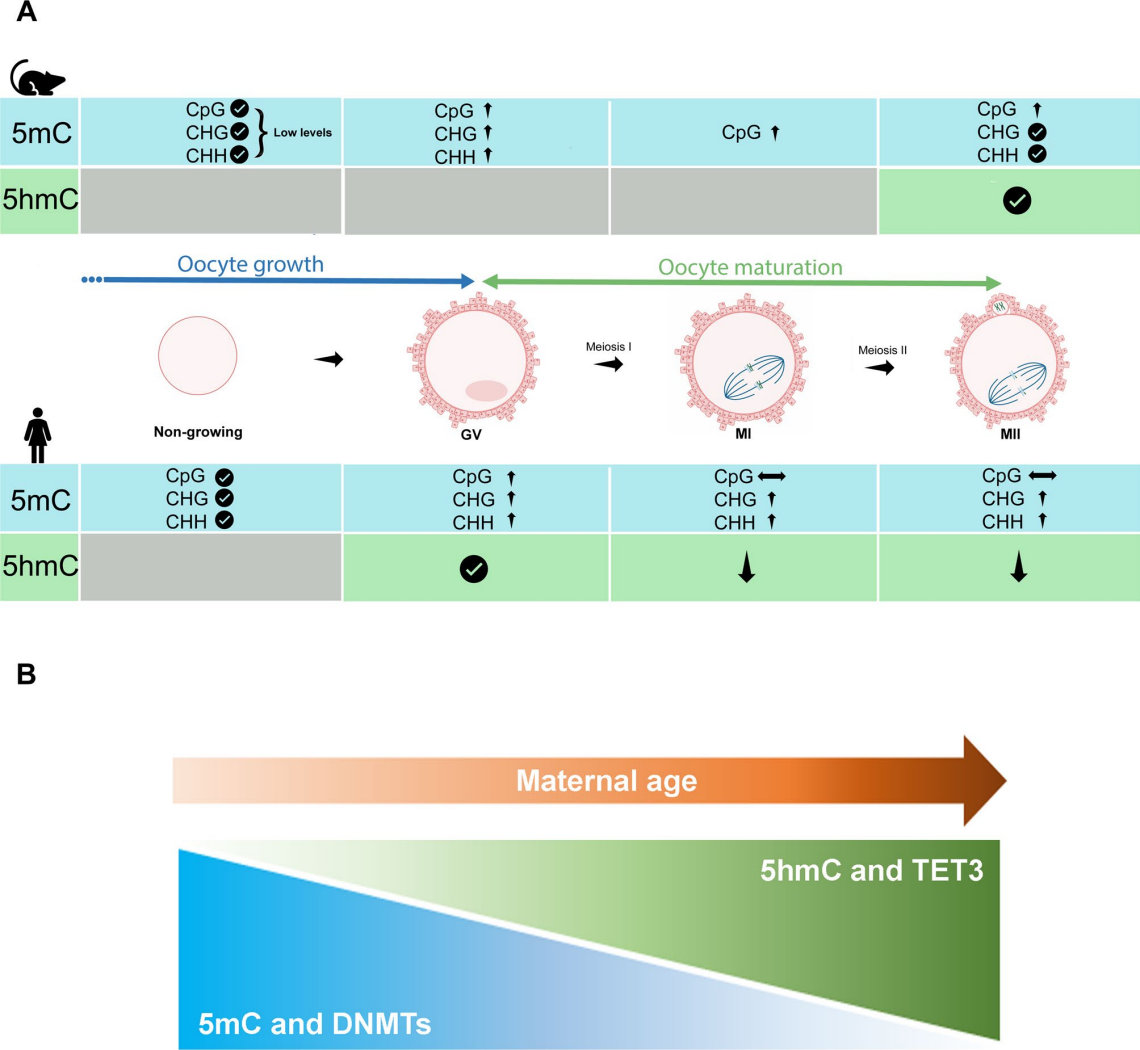
(**A**) Overview of the dynamics of DNA methylation (5-mC) and DNA hydroxymethylation (5hmC) levels throughout oocyte growth and maturation in human and mouse. (**B**) Overview of the 5mC, DNMTs, 5hmC and TET3 expression levels with advanced maternal age, based on the following publications [98, 99, 107]. The upward arrow represents increased levels, the downward arrow represents decreased levels, in DNA methylation or hydroxymethylation. The horizontal arrow represents constant levels of DNA methylation and hydroxymethylation. Grey background represents absence of information. GV- Germinal vesicle oocyte; MI- Metaphase I oocyte; MII- Metaphase II oocyte; 5mC- 5-methylcytosine; 5hmC- 5-hydroxymethylcytosine

Regarding oocyte maturation, Smallwood and collaborators reported levels of 0.5% highly methylated CpGs in day 5 mouse oocytes, 11.3% in day 20 GV oocytes and 15.3% in ovulated metaphase II oocytes [45] (Fig. 3A). They also reported 1,600 fully methylated CGIs in mature MII oocytes, 89 of which were unmethylated in GV oocytes. A comparison with sperm cells revealed significant differences, with only 185 methylated CpG islands detected in spermatozoa, 58 of these exclusive to sperm. Furthermore, CGIs acquired methylation at different rates during oocyte growth, with methylated CGIs preferentially located within active transcription units, supporting a transcription-dependent methylation mechanism. Additionally, methylated CGIs were mainly found in intragenic regions with lower GC content and CpG density. However, these methylation differences did not significantly impact the expression levels of genes associated with methylated CGIs.

Thus, the unique methylation pattern of the mouse oocyte is characterized by a bimodal DNA methylation profile, encompassing both low and high levels of DNA methylation [46]. Unlike sperm, which exhibit high global methylation, oocyte methylation is primarily associated to actively transcribed regions, particularly gene bodies. The distinctive methylation landscape of oocytes, comprising alternating hyper- and hypomethylated domains, has also been observed in mouse oocytes retrieved after superovulation or after in vitro follicle culture (IFC). These conditions did not affect methylation at imprinted germline Differentially Methylated Regions (DMRs) [47]. However, some gene-specific differences were noted in IFC and in oocytes from prepubertal and sexually mature females. On the other hand, work by Melissa Mann’s lab showed that superovulation perturbs methylation at multiple imprinted loci in a dose-dependent manner in the preimplantation embryo, affecting both maternally and paternally inherited alleles [48]. However, imprinted methylation acquisition was not perturbed in mouse oocytes by superovulation, suggesting that superovulation may impact early embryogenesis instead [49]. Altogether, these studies indicate that superovulation does not impair the establishment of imprinted methylation in oocytes, although it may compromise the stability or maintenance of these marks after fertilization.

In addition, studies have shown that specific histone modifications regulate DNA methylation in mouse oocytes. In fact, histone H3K36me3 and H3K36me2vmodifications, mediated by SETD2, and NSD1 methyltransferases, respectively, provide the chromatin platform essential for guiding DNMT3A-dependent *de novo* DNA methylation in mouse oocytes. Furthermore, it has been shown that the two histone marks contribute to distinct levels of DNA methylation: H3K36me2, which is broadly distributed across intergenic and weakly transcribed regions, is associated with moderate methylation levels, while H3K36me3, which marks actively transcribed regions, is linked to high levels of DNA methylation [50]. H3K36me3 is established early during oocyte growth and increases in parallel with transcriptional activity and DNA methylation, remaining enriched until the MII stage. Additionally, maternal depletion of SETD2 impairs oocyte maturation and leads to developmental arrest at the one-cell stage following fertilization [51]. Proper DNA methylation at CGIs in oocytes requires the removal of protective H3K4me2/3 marks, a process mediated by the demethylases KDM1A and KDM1B, while the acquisition of H3K36me3 at these sites in growing oocytes facilitates methylation establishment [52].Together, DNA methylation and histone modifications control chromatin accessibility and packaging, allowing gene activation or repression.

High levels of non-CpG methylation have been also observed in GV oocytes but not in sperm, blastocysts or embryonic stem (ES) cells [42]. GV oocytes show five times more non-CpG methylation compared to non-growing oocytes (3.2% vs. 0.6%, respectively) [43] (Fig. 3A). This accumulation is driven by the *de novo* methyltransferase DNMT3A and its cofactor DNMT3L, as their absence leads to a global reduction in both CG and non-CG methylation. In contrast, DNMT3B appears to be non-essential in these circumstances. DNMT1 deficiency also results in a modest decrease in CG methylation in oocytes [43]. The high levels of non-CpG methylation observed in oocytes likely result from the elevated expression of the *de novo* DNA methyltransferases during these stages. More recently, it was shown that non-CpG methylation occurs in both SN and NSN oocytes at a frequency of about 4% [53]. Non-CpG methylation in oocytes is lost during early embryonic divisions, as it is not maintained after DNA replication. By the blastocyst stage, its levels are similar to those in post-implantation embryos [54].

Regarding the establishment of germline maternal imprinting marks, these are progressively established during oogenesis and are protected from demethylation in early embryonic development, by zygotic factors such as ZFP57, TRIM28, UHRF1 and DNMT1 [34]. Maternally methylated imprinted genes, such as *Peg3*, *Snrpn*, *Gnas* and *Kcnq1ot1*, exhibit full ICR methylation in mature MII mouse oocytes [42–44, 55]. However, Li and colleagues observed that CpG islands near the maternally imprinted genes *Ndn*, *Magel2*, *Mkrn3*, *Peg12*, and *Igf2* remained completely unmethylated in MII oocytes, whereas others near *Peg3*, *Kcnq1* and *Snrpn* became fully methylated between GV and MII oocyte stages [56]. While most studies focus on CG methylation, it´s worth noting that non-CG methylation has also been detected in maternally methylated ICRs from GV oocytes [42, 43, 54]. Non-CpG methylation was first described in mouse oocytes at individual genes such as *Peg1/Mest* and *Nf1* genes [57, 58] and later detected in several other imprinted regions [54].

Uysal and colleagues investigated the spatial and temporal expression of the DNA methyltransferases DNMT1, DNMT3A and DNMT3B in GV and MII mouse oocytes. Their findings revealed that DNMT1 is primarily localized to the cytoplasm and more abundant in MII oocytes, whereas DNMT3A and DNMT3B were localized in the nucleus at both GV and MII maturation stages [59]. At the transcriptional level, Lucifero and collaborators reported an increase in *Dnmt1o*,* Dnmt3a*, *Dnmt3b* and *Dnmt3L* expression during oocyte growth, which was correlated with oocyte diameter [60]. Several studies have demonstrated that CpG and non-CpG methylation in mouse oocytes depends on DNMT3A and DNMT3L [42, 45], and that DNMT1 might play a role in *de novo* methylation in non-dividing oocytes, of CpGs that remained hemi-methylated [43].

Remarkably, Dnmt3L KO mouse oocytes were found to be almost completely devoid of methylation [42]. The absence of *Dnmt3a* or *Dnmt3L* resulted in a global reduction in both CG and non-CG methylation, affecting intragenic and intergenic regions as well as repetitive elements [43]. Furthermore, DNMT3A or DNMT3L null oocytes failed to acquire methylation imprints during oocyte development, leading to defective maternal imprinting marks and impaired embryonic development [61, 62]. Hence, DNMT3A and its co-factor DNMT3L are essential DNA methyltransferases responsible for *de novo* DNA methylation in oocytes at both CpG and non-CpG sites.

To summarize, chromatin accessibility changes at the onset of oocyte growth coincide with increased transcriptional activity, while DNA methylation increases more dramatically later in oocyte development [63]. These findings indicate an accumulation of DNA methylation during oocyte growth, affecting both CpG and non-CpG sites, and is closely associated with active transcription and chromatin accessibility. Although the majority of DNA methylation is globally erased following fertilization, the late-stage methylation changes observed in oocytes are likely to have important regulatory implications as oocyte-acquired DNA methylation may influence chromatin organization by shaping histone modifications and establishing transcriptionally repressive domains, as is the case of the topological associated domains (TADs) observed in embryos that show different levels of CpG methylation [64]. Additionally, DNA methylation is critical for the silencing of transposable elements in the oocyte, some of which may partially escape global demethylation and impact genomic stability or transcription in the embryo. Taken together, these findings support the notion that even transient methylation acquired in the oocyte can contribute to epigenetic inheritance and developmental programming.

### DNA methylation during oocyte maturation – human studies

Several studies have investigated the dynamics of DNA methylation during human oocyte maturation. Yu and colleagues, using single-cell whole-genome bisulfite sequencing (sc-WGBS), observed that the three maturation stages, GV, MI and MII, had an average CpG methylation level of approximately 50%, with no significant differences between stages [65]. Similar levels of CpG methylation were also reported by other authors [66–69], indicating that CpG methylation is re-established during oocyte growth, being completed by the GV stage (Fig. 3A). Guo and colleagues reported that hypermethylated regions in human oocytes are strongly enriched in transposable elements, similar to what is observed in sperm, potentially repressing their transcription and deleterious activity [66]. In contrast, hypomethylated regions are enriched in high-density CpG promoters and CpG islands. Additional research conducted by Yan and colleagues, using single-cell technology, also demonstrated that *de novo* methylation occurs in growing oocytes (GO) and is closely associated with chromatin accessibility and gene transcription, both at gene bodies and in maternally imprinted regions [69]. They observed strong heterogeneity in DNA methylation levels among growing oocytes, concomitantly with high heterogeneity in chromatin accessibility; however, fully-grown (FGO), MI and MII oocytes were more homogeneous and presented a higher level of methylation. Guo and colleagues observed that, in MII oocytes, DNA methylation levels of gene bodies were higher than those in intergenic regions [66]. Additionally, Ye and collaborators reported that exons, introns and 3’UTR regions displayed higher CpG methylation levels than CpG islands, promoters and 5’UTRs, in mature oocytes [68].

Several authors have explored the dynamics of DNA methylation at imprinted genes during oocyte maturation. Khoureiry and colleagues investigated the methylation status of the KCNQ1OT1 differentially methylated region (KvDMR1) in human oocytes at different stages of oocyte maturation. Their findings showed a progressive increase in KvDMR1 methylation levels during oocyte maturation, suggesting that *de novo* methylation of KvDMR1 coincided with the progression to meiosis II [70]. However, in the study conducted by Geuns and collaborators, these differences throughout oocyte maturation were not observed, suggesting that maternal methylation imprints were already established at the GV stage [71]. Additionally, the study of *DLK1* and *GTL2* IG-DMR, a paternally methylated DMR, showed complete demethylation at all maturation stages, indicating that erasure of previous methylation imprints occurred before the GV stage [72]. Complete methylation of other DMRs such as *PEG1*, *LIT1*, *SNRPN*, *ZAC* and *PEG3* was also observed during different stages of the oocyte maturation [73–76].

Regarding non-CpG methylation, levels significantly increased during the oocyte maturation process, reaching their highest levels in MII oocytes (Fig. 3A), potentially directed by the increased activity of DNMT3B in mature oocytes [65, 77]. Remarkably, non-CpG DMRs were enriched at transposable elements and correlated with expression of genes nearby [77]. Furthermore, genome-wide non-CpG methylation level around the transcriptional start sites (TSSs) increased in growing oocytes but decreased in mature oocytes, indicating a dynamic remodelling process [69]. The observed increase in non-CpG methylation during oocyte maturation may influence gene expression, generally leading to repression across most genomic regions, while promoting transcription when occurring within gene bodies such as introns, exons, and 3′UTRs. These findings highlight the potential regulatory role of non-CpG methylation in oocyte development and underscore the need for further investigation beyond CpG sites [54].

Ye and collaborators observed that the global levels of CpG methylation in in vivo and in vitro matured oocytes were similar, whilst non-CpG methylation was significantly lower in in vitro matured MII oocytes [68]. Regarding imprinted genes, abnormal methylation at the DLK1-GTL2 imprinted region was observed in in vitro matured oocytes [74]. Al-Khtib and collaborators reported nearly complete methylation at the KvDMR1 in in vitro matured oocytes and after vitrification [78], although Shi and colleagues reported abnormal methylation in 8.8% of MII oocytes after in vitro maturation [76]. Several authors also analysed the methylation profile of *H19* region, consistently observing its unmethylated state in GV and MII oocytes [75, 76, 78, 79]. However, Borghol and colleagues noted that oocytes arrested in maturation or subjected to in vitro maturation showed hypermethylation at the *H19* DMR, raising caution over in vitro maturation technique [79].

Regarding the expression of DNA Methyltransferases, Yan and colleagues observed that *DNMT1* and *DNMT3A* were expressed in GO as well as in oocytes from all maturation stages. In contrast, *DNMT3B* exhibit significant expression exclusively in FGO, MI and MII oocytes [69]. This observation was further corroborated by Okae and colleagues [67], suggesting a potential pivotal role for DNMT3B in *de novo* DNA methylation during human oocyte growth. Petrussa and colleagues also evaluated the DNMT levels in oocyte maturation using immunostaining. Their findings indicated that DNMT1 was consistently present throughout all maturation stages of human oocytes. Notably, in GV oocytes, the DNMT1 had a nuclear localization, whereas in MI and MII oocytes, it displayed a cytoplasmic localization. This suggests that DNMT1 proteins are sequestered within the oocyte nucleus, facilitating the maintenance of methylation during oocyte maturation, while DNMT3A and DNMT3B were consistently localized in the oocyte cytoplasm across all the maturation stages but were notably absent from the nucleus [80]. In a study conducted by our research group, we observed that *DNMT1* exhibited high expression throughout oocyte maturation, with a marked upregulation in MII oocytes. Conversely, DNMT3A and DNMT3B were expressed across all maturation stages, although their expression levels were significantly lower compared to *DNMT1*. Notably, *DNMT3L* expression was not observed at any stage of oocyte maturation [81].

### DNA hydroxymethylation in oocyte

DNA methylation has been extensively studied in oocytes from both humans and mice. However, studies addressing DNA hydroxymethylation and TET enzymes remain scarce, particularly in the context of oocyte maturation. One of the few studies addressing DNA hydroxymethylation in human GV, MI and MII oocytes performed immunostaining and showed that 5hmC signal was localized within the oocyte nucleus during oocyte maturation, with a slight decrease in 5hmC fluorescence intensity as maturation progressed [82] (Fig. 3A). Yan and colleagues studied 5hmC at base resolution in MII oocytes using APOBEC-Coupled Epigenetic Sequencing (ACE-Seq). This comprehensive analysis revealed that 5hmC levels in MII oocytes were approximately 1.6%, increasing to 2.13% in the zygotic maternal pronucleus, whereas in sperm, the levels were 0.67% rising to ~ 9% in the paternal pronucleus. Among various genomic elements examined, CGIs showed the highest levels of DNA hydroxymethylation, in contrast to gene bodies, intergenic and repetitive elements, which displayed relatively low levels of 5hmC in the oocyte. Additionally, TET-dependent hyper-hydroxymethylated differentially methylated regions (hmDMRs) in the maternal pronucleus revealed a 5hmC percentage of approximately 3.7% in MII oocytes [83]. While this study provided valuable insights into DNA hydroxymethylation levels in the oocyte, it’s important to note that only two MII oocytes were analysed. To gain a more comprehensive understanding of how DNA hydroxymethylation changes throughout oocyte maturation, further investigations involving a larger number of oocytes at different maturation stages would be an asset.

In mice, it has been shown that TET3, one of the three enzymes of the TET family of dioxygenases, is highly expressed in oocytes [36, 37, 84]. Additionally, Wossidlo and collaborators reported that downregulation of *Tet3* in the pronuclear zygote resulted in decreased 5hmC and increased 5mC in the paternal pronucleus, suggesting a failure to convert 5mC to 5hmC [37]. In a subsequent study, the authors showed that triple knockdown of all three TET enzymes completely prevented blastocyst development, with the majority of the embryos arresting at the two-cell stage [85], possibly due to failure in the activation of the paternal genome [86]. In fact, we observed that, amongst the TET-family enzymes involved in the active DNA demethylation process, *TET3* was the most abundantly expressed across all stages of oocyte maturation. Additionally, *TET2* was also expressed throughout maturation, while *TET1* was not expressed at any stage [81].

Gu and collaborators previously demonstrated that maternal deletion of *Tet3*, albeit not affecting oocyte development, maturation and fertilization, resulted in impaired reprogramming of the paternal genome in the early zygote. Embryos lacking maternal *Tet3* exhibited a reduced rate of full-term development and a high incidence of morphological abnormalities. Importantly, they have also observed that homozygous deletion of *Tet3* led to neonatal lethality [36].

Moreover, *Tet1* deletion has been associated with subfertility, with a decrease in the number of fully grown oocytes due to apoptosis of meiotically-arrested oocytes, deficient demethylation and decreased expression of a subset of meiotic genes [87]. Additionally, *Tet1* deficiency in mouse oocytes resulted in hypermethylation of a subset of imprinting control regions (ICRs) and consequent aberrant expression of imprinted genes [88]. Furthermore, *Tet1* deficiency in mice has been linked with accelerated reproductive failure with age and led to premature ovarian failure [89].

## The ageing oocyte – an epigenetic perspective

Ageing is a complex, multifactorial process of molecular and cellular decline that affects every cell and organ, ultimately leading to physiological deterioration that impairs normal biological functions [90]. The mouse is commonly used as a model for human physiologic ageing, with studies demonstrating similarities in female reproductive ageing between mouse and human [91].

Female germ cell meiosis is a long and complex process, characterized by two consecutive cell divisions - meiosis I and II - as described above. The success of this maturation process has direct implications for oocyte quality, required for fertilization and subsequent embryo development [92]. Advanced maternal age leads to a progressive decline in both oocyte number and quality, as well as a decrease in developmental potential [93, 94]. Poor oocyte quality is correlated with compromised genetic integrity and epigenetic changes during the oocyte ageing process [95, 96]. However, the underlying epigenetic mechanisms contributing to the decline in oocyte quality during postovulatory oocyte ageing and advanced maternal age still requires further investigation. Here, we address how epigenetics might play a role in these ageing processes.

Studies have compared expression profiles of MII oocytes between young and older females to assess the effects of maternal ageing. Based on microarray analysis, Hamatani and collaborators found that, amongst approximately 11,000 genes with detectable transcripts in MII oocytes, about 5% (530 genes) were affected by maternal ageing. Interestingly, among the differentially expressed genes, several were involved in chromatin structure and DNA methylation [97]. Moreover, Yue and colleagues studied the expression of DNMTs in MII oocytes and evaluated the global DNA methylation levels in oocytes and pre-implantation embryos from young (6–8 weeks) and old (35–40 weeks) mice. Their findings indicate that DNA methylation levels in oocytes and preimplantation embryos significantly decreased with increased maternal age [98]. In addition, DNMTs expression in MII oocytes of older age were lower, and the pregnancy rates in older female mice were diminished [98]. Reduced DNMT transcript and protein levels have been observed in MII oocytes from older females when compared to young females [98, 99]. DNMTs expression in ageing mouse ovaries have also been documented to be decreased [100]. Furthermore, the effects of maternal ageing on the localization of DNMT3a and DNMT3b were explored, revealing that maternal ageing affects cytoplasmic-to-nuclear trafficking during meiosis [101]. Altogether, these studies suggest that the decreased reproductive potential associated with maternal ageing may be attributed to defects in global DNA methylation or *DNMTs* expression during oogenesis and preimplantation embryo development. However, it is important to note that this decline of reproductive potential associated to reproductive aging is multifactorial and could involve other factors, including oxidative stress, mitochondrial defects, telomere shortening, meiotic chromosome segregation errors and genetic alterations, which collectively impair oocyte quality [102].

The methylome of the ageing oocyte remains relatively unexplored. Castillo-Fernandez and colleagues conducted a comprehensive profiling of both the transcriptome and DNA methylome of individual GV oocytes obtained from young and older mice undergoing natural ovulation [103]. The transcriptome of oocytes derived from advanced age females showed increased heterogeneity and variability in gene expression. Furthermore, while there was a slight reduction in total CpG methylation, an increase in non-CpG methylation was observed in the advanced age group. The DMRs of imprinted genes maintained appropriate methylation levels irrespective of age. This observation aligns with the findings of other authors who investigated *Snrpn* and *Kcnq1ot1* germline DMRs in mouse oocytes, reporting normal methylation acquisition regardless of age, with no impact on methylation maintenance up to the blastocyst stage following fertilization of oocytes from aged females [104]. Several studies have also analysed methylation patterns of different imprinted genes in oocytes during postovulatory ageing, showing that imprinted DNA methylation patterns do not appear to be vulnerable to postovulatory age. Lucifero and colleagues showed the persistence of the hypermethylated pattern of *Peg1* in fresh oocytes through postovulatory ageing [60]. In contrast, Imamura and colleagues observed that prolonged in vitro culture led to the loss of methylation of *Peg1* in oocytes [58]. Liang and colleagues further investigated methylation patterns of imprinted genes in mouse oocytes during the postovulatory ageing process. Initially, they assessed the methylation status of *Peg1* and *Snrpn* in postovulatory oocytes, both aged in vivo and in vitro, at various time points. Results indicated that *Snrpn* maintained a complete methylation pattern in oocytes during the initial hours, but after 29 h post hCG injection, some oocytes exhibited methylation loss. However, *Peg1* showed no demethylation across all aged groups [105]. Subsequently, they expanded their analysis to investigate the DNA methylation patterns of imprinted genes in oocytes derived from offspring originating from postovulatory aged oocytes. They found that methylation patterns at DMRs of *Peg3*, *Snrpn*, *Peg1* and *H19* in oocytes from aged-oocyte offspring were predominantly normal, with only a small fraction of oocytes showing aberrant methylation in *Peg3* DMR [106]. Despite the negative impact of postovulatory oocyte aging on reproductive outcomes, it does not seem to disrupt the acquisition of methylated imprints in oocytes.

DNA methylation is one central epigenetic mechanism involved in critical developmental processes and has been implicated in ageing [91]. Nonetheless, the impact of ageing on DNA demethylation pathways in oocytes remains unexplored. Qian and colleagues studied DNA demethylation marks, including 5hmC, 5fC and 5caC, at the cellular level, as well as TET enzymes expression in oocytes from both natural ageing and accelerated oocyte ageing conditions, specifically premature ovarian ageing [107]. First, the authors examined naturally ageing oocytes and observed an increase in 5hmC level correlated with the progression of natural ageing (Fig. 3B). They identified *Tet3* as the predominant player in DNA demethylation pathway, with *Tet3* expression increasing with age. Furthermore, they noted an increase in 5caC levels and a decreased in 5fC with age. Intriguingly, in accelerated ageing, 5fC levels increased, while, similarly to natural ageing oocytes, 5hmC levels also increased. The aberrant expression pattern of *Tet3* was even more pronounced in the accelerated ageing condition than natural ageing oocytes, suggesting a potentially significant role for *Tet3* in this accelerated aging process.

Thus, the epigenetic status of oocytes is dynamic, undergoes changes with ageing that could potentially impact the developmental potential of embryos. Limited evidence exists regarding whether *TET* expression in oocytes is modulated by ageing. One plausible mechanism that we proposed is the diminished expression of *DNMTs* and the observed reduction in methylation levels in oocytes with advanced age, possibly stemming from increased demethylation, with *Tet3* emerging as a crucial enzyme in oocyte ageing (Fig. 3B). Alterations in these epigenetic regulators, DNMT and TET enzymes. may directly contribute to epigenetic changes in aged oocytes. Understanding these dynamics could provide novel perspectives for future research and potentially add new strategies aiming at improving female reproductive outcomes across ageing.

## Conclusions and future perspectives

Oocyte maturation is a complex process regulated by numerous molecules and signaling pathways, involving multiple steps. The current focus on studying oocytes quality and its application in clinical practice arises from frequent failures in fertilization, early embryo development, and pregnancy success during in vitro fertilization protocols.

While significant progress has been made in understanding the nature of oocyte maturation over the past decade, there remains a paucity of studies comparing the three stages of maturation – an essential aspect for comprehending oogenesis. Most researchers primarily focus on analysing DNA methylation patterns, with limited exploration of the role of DNA hydroxymethylation in oocyte maturation. Future research directions could involve conducting comprehensive, genome-wide analysis of 5hmC levels in oocytes at the three stages of maturation and correlating these levels with the respective transcriptional levels, particularly in relation to TET enzyme activity. The emerging understanding of epigenetics in oocyte maturation presents a significant challenge to clinical approaches, while also offering new opportunities for addressing human infertility. Additionally, the increasing prevalence of oocyte failure associated with female reproductive ageing presents a growing health concern. The challenges posed by advanced maternal age and age-related fertility decline underscore the need to shift focus towards investigating epigenetic changes not only in oocytes but also in follicular granular cells. Understanding the mutual communication and physical interactions between these cells is crucial for advancing reproductive medicine.

## Data Availability

No datasets were generated or analysed during the current study.

## Abbreviations

GV: Germinal vesicle
GVBD: Germinal vesicle breakdown
MAPK: Mitogen–activated protein kinase
MI: Metaphase I
MII: Metaphase II
CSF: Cytostatic factor
MPF: Maturation promoting factor
NSN: Non–surrounded nucleolus
SN: Surrounded nucleolus
cAMP: Cyclic adenosine monophosphate
GC: Granulosa cells
LH: Luteinizing hormone
CC: Cumulus cells
CPE: Cytoplasmic polyadenylation element
UTR: Untranslated region
mtDNA: Mitochondrial DNA
ER: Endoplasmic reticulum
GA: Golgi apparatus
ZP: Zona pellucida
cGMP: Cyclic guanosine 3’,5’–monophosphate
PDE3A: Phosphodiesterase 3 A
5mC: 5–methylcytosine
5hmC: 5–hydroxymethylcytosine
5fC: 5–formylcytosine
5caC: 5–carboxylcytosine
CpG: Cytosine–phosphate–guanine
PGCs: Primordial germ cells
ICR: Imprinting control region
LINE: Long interspersed nuclear element
SINE: Short interspersed nuclear element
LTRs: Long terminal repeats
NGO: Non–growing oocytes
GO: Growing oocytes
FGO: Fully grown oocytes
ES: Embryonic stem cells
DMRs: Differentially methylated regions
IFC: In vitro follicle culture
CGI: CpG islands
TSS: Transcription start site
TTS: Transcription termination site
